# Quality Variation Patterns and Predictive Modeling of Fermented Soybean Whey-Based Tofu Under Cold-Chain Conditions Using Kinetic and Machine Learning Approaches

**DOI:** 10.3390/foods15173147

**Published:** 2026-09-04

**Authors:** Dan Zhao, Zhanrui Huang, Hao Chen, Liangzhong Zhao, Xiaohu Zhou, Xiaojie Zhou, Liu Fan, Fengwu Li

**Affiliations:** 1 College of Economics and Management, Shaoyang University, Shaoyang 422000, China; 2 Hunan Provincial Key Laboratory of Soybean Products Processing and Safety Control, Hunan Engineering Research Center of Green Processing and Equipment of Hunan-Style Food, Shaoyang University, Shaoyang 422000, China; 3 Yanjinpuzi Food Co., Ltd., Liuyang 410329, China

**Keywords:** fermented soybean whey-based tofu, cold-chain transportation, quality deterioration, microbial kinetics, hybrid prediction model, machine learning

## Abstract

Pre-packaged fermented soybean whey-based tofu (FSW-tofu) was stored under dynamic temperature conditions (4–20 °C) that simulated typical supermarket and e-commerce cold-chain transport modes. Changes in total viable count (TVC), psychrophilic bacterial count (PBC), hardness, springiness, chewiness, and water-holding capacity were monitored over 35 d, and a hybrid prediction model integrating mechanistic kinetics with machine learning was established. Results indicated that both temperature fluctuation amplitude and frequency significantly affected microbial proliferation and textural degradation. Under the e-commerce mode, exposure to 20 °C accelerated the TVC, reaching 5 lg CFU/g at 17 d, earlier than under the supermarket mode (27 d). However, the sustained low-temperature stress in the supermarket mode caused more profound degradation of the protein gel network, leading to more severe textural deterioration at the equivalent TVC threshold. The Baranyi–Roberts–Ratkowsky non-isothermal growth model and quality response functions served as the base framework, while random forest and gradient boosting trees were used for residual correction, yielding a coupled mechanistic-data-driven model. Independent validation yielded R^2^ > 0.89 and relatively low RMSE, confirming the model’s good generalization and predictive accuracy. This approach combines mechanistic interpretability with machine learning accuracy to provide a rapid assessment tool for the cold-chain quality management of FSW-tofu.

## 1. Introduction

Fermented soybean whey-based tofu (FSW-tofu) is a traditional soybean product that is curdled using fermented soybean whey, which is itself derived from the whey squeezed out during tofu processing. Owing to its fine texture, rich soybean aroma, and absence of chemical additives, FSW-tofu aligns well with current consumer trends toward “clean-label” products and the industrialization of traditional foods; consequently, its market share has grown steadily in recent years [1,2]. Compared with tofu made with coagulants such as Ca^2+^ and Mg^2+^, FSW-tofu offers distinct advantages in retaining natural nutrients, including soybean isoflavones and oligosaccharides. However, its high moisture content (typically > 80%) and abundant protein (approximately 6–8%) provide an ideal nutritional basis for microbial growth [3,4]. More importantly, FSW-tofu itself has a slightly acidic pH (5.0–5.5), a range that is particularly favorable for the growth of most spoilage bacteria (e.g., *Pseudomonas* spp. and *Enterobacteriaceae*). In the absence of chemical preservatives, FSW-tofu is highly susceptible to quality deterioration during post-processing storage and distribution, rendering its dependence on and sensitivity to cold-chain transportation significantly greater than that of other soybean products [5,6].

The quality deterioration of FSW-tofu is a complex process that is dominated by microorganisms and influenced by multiple interacting factors. Extracellular proteases produced by microbial metabolism can degrade β-conglycinin (7S) and glycinin (11S) within the soybean protein gel network, disrupting the hydrophobic interactions, disulfide bonds, and hydrogen bonds that maintain gel structure, which directly leads to decreased hardness and loss of springiness [7]. Concurrently, polar groups exposed during protein degradation alter the charge distribution of the gel network, weakening its physical water-holding capacity (WHC), which manifests as reduced WHC and increased water loss [8]. In addition, free radicals generated from lipid oxidation may further induce protein cross-linking or cleavage, thereby exacerbating textural deterioration [9]. The rates of all these deteriorative reactions are positively correlated with temperature. However, actual cold-chain transportation involves multiple stages, including factory cooling, refrigerated transport, distribution center storage, retail refrigeration, and last-mile delivery, with substantial temperature variations across these stages (4–20 °C) [10,11]. Thus, FSW-tofu products are continuously exposed to dynamic, non-isothermal temperature conditions, and these fluctuations render microbial growth kinetics highly complex: elevated temperatures not only increase the specific growth rate but also shorten the lag phase, while subsequent cooling cannot fully “reverse” the textural deterioration incurred during prior high-temperature exposure [11,12]. Traditional predictive models based on constant temperature conditions or conventional quality evaluation methods are incapable of accurately capturing the actual deterioration progression under such temperature memory effects.

Predictive microbiology has provided effective theoretical tools for assessing food quality under dynamic temperature conditions, and the field has undergone continuous evolution from classical models to intelligent algorithms [13]. At the classical modeling stage, researchers have primarily employed primary models such as Baranyi–Roberts and Logistic to describe changes in microbial counts over time, combined with secondary models like the Ratkowsky square-root model, Arrhenius-type equations, or the Huang square-root model to depict the effect of temperature on growth rates. These approaches have been successfully applied to predict non-isothermal growth in perishable foods such as meat and aquatic products [14]. For instance, Sun et al. [15] used a modified Baranyi model to simulate the dynamic growth of microorganisms in tilapia under cold-chain temperature fluctuations, while Huang et al. [16] developed a growth kinetic model for *Clostridium perfringens* in cooked beef during cooling by integrating the Runge–Kutta method and Monte Carlo simulation. In the subsequent intelligent algorithm-assisted stage, researchers have introduced machine learning methods (including random forest, gradient boosting trees, support vector regression, and artificial neural networks) into predictive frameworks [17,18]. Machine learning can automatically capture high-order interactions and nonlinear responses, compensating for the systematic biases of classical kinetic models when faced with complex and non-stationary temperature profiles, thereby further expanding the application boundaries of predictive models [18,19].

Nevertheless, research on predictive modeling specifically for fresh tofu, and particularly for FSW-tofu, remains significantly lagging. A few studies have examined the quality changes in FSW-tofu at constant temperatures; for example, Huang et al. [20] investigated the refrigerated quality of FSW-tofu at 4 °C, and Zeng et al. [21] reported the retarding effect of ε-polylysine on the increase in total viable count (TVC) in pre-packaged FSW-tofu. However, these studies were all confined to isothermal conditions. To date, no systematic study has reported the microbial growth patterns, quality response characteristics, or predictive models for FSW-tofu under realistic dynamic temperature fluctuations during cold-chain transportation, nor has there been any methodological exploration that combines classical predictive microbiology frameworks with machine learning approaches for the FSW-tofu system. This gap not only limits the academic understanding of the mechanisms driving quality deterioration in FSW-tofu but also hinders the scientific management of quality risks during cold-chain processes in the industry.

Therefore, pre-packaged FSW-tofu was used as the research object in this study, and dynamic temperature fluctuations (4–20 °C) from two typical cold-chain transport modes (supermarket and e-commerce) were simulated. The changes in TVC, psychrophilic bacterial count (PBC), WHC, and textural properties (hardness, springiness, chewiness) during storage were systematically analyzed. On this basis, the Baranyi–Roberts primary model and the Ratkowsky secondary model were coupled to construct a non-isothermal microbial growth kinetic model, and a mechanism-driven prediction framework for quality responses was established. Furthermore, a composite machine learning model combining random forest and gradient boosting trees was introduced for residual correction of the mechanistic predictions, ultimately yielding a comprehensive dynamic prediction model for the cold-chain quality of FSW-tofu that offers both mechanistic interpretability and high predictive accuracy. The Baranyi–Roberts model was selected for its ability to characterize the lag-phase perturbation caused by temperature fluctuations, while the Ratkowsky model was chosen for its robust description of temperature dependence on microbial growth rates. Random forest and gradient boosting trees were adopted for their proven capability to capture nonlinear interactions and resistance to overfitting with small-to-medium datasets. This study aims to elucidate the core drivers of quality deterioration in FSW-tofu under different cold-chain transportation modes and to provide scientific evidence and technical support for optimizing cold-chain processes, establishing quality monitoring checkpoints, and rapidly assessing quality deterioration at the retail level

## 2. Materials and Methods

### 2.1. Materials and Reagents

Canadian non-GMO soybeans (protein content 38%) were purchased from the Wanyue Import and Export Trade Co., Ltd. (Yueyang, China). Fermented soybean whey (total acid content: 4.49 ± 0.1 g/L; protease content: 9.51 ± 0.2 U/mL) was obtained from the Hunan Provincial Key Laboratory of Soybean Products Processing and Safety (Shaoyang, China). Plate count agar (PCA) was purchased from Guangdong Huankai Microbial Technology Co., Ltd. (Guangzhou, China). All other chemical reagents were purchased from Sinopharm Chemical Reagent Co., Ltd. (Shanghai, China).

### 2.2. Equipment

The following equipment was used: soymilk integrated equipment (SJJ-20, Kangdeli Intelligent Technology (Zhejiang) Co., Ltd., Jiaxing, China); texture analyzer (TA1, Lloyd Instruments Ltd., Bognor Regis, UK); homogenizer (Sicentz-150, Ningbo Scientz Biotechnology Co., Ltd., Ningbo, China); thermostatic incubator (BOI-250, Shanghai Santeng Instruments Co., Ltd., Shanghai, China); reverse-pressure high-temperature sterilizer (ZY-50S, Zhejiang Xinfeng Medical Equipment Co., Ltd., Shaoxing, China); and centrifuge (TGL18S, Changsha Yingtai Instrument Co., Ltd., Changsha, China).

### 2.3. Sample Preparation

The FSW-tofu was prepared according to the following procedure. Soybeans were soaked in deionized water (1:3, *w*/*v*) at 25 °C for 8 h, then ground with water (1:5, *w*/*v*) using a soymilk-integrated equipment (SJJ-20). The resulting slurry was filtered through a 200-mesh sieve, and the filtrate was boiled at 105 °C for 5 min. Fermented soybean whey was added to the boiled soymilk at a ratio of 1:4 (*v*/*v*) at 85 °C with gentle stirring until coagulation was complete. The curd was transferred into molds, pressed for 2 h under 0.5 kPa, and cooled to obtain the FSW-tofu [1].

The FSW-tofu was cut into blocks (250 g; 7.0 × 10.0 × 4.0 cm) and aseptically packaged into sterile polyethylene containers, and randomly assigned to six experimental groups (350 pieces in each group). Each container was supplemented with approximately 90 g of an immersion solution containing 0.2% chitosan oligosaccharide (COS) (pH 5.0, 2 mg/mL, *w*/*w*) [6,22]. After sealing and packaging, the tofu samples were quickly subjected to reverse-pressure sterilization (85 °C, 0.1 MPa gauge pressure, 30 min), cooled to room temperature, and then refrigerated.

### 2.4. Sample Dynamic Storage Temperature and Time Setting

**Figure 1:**
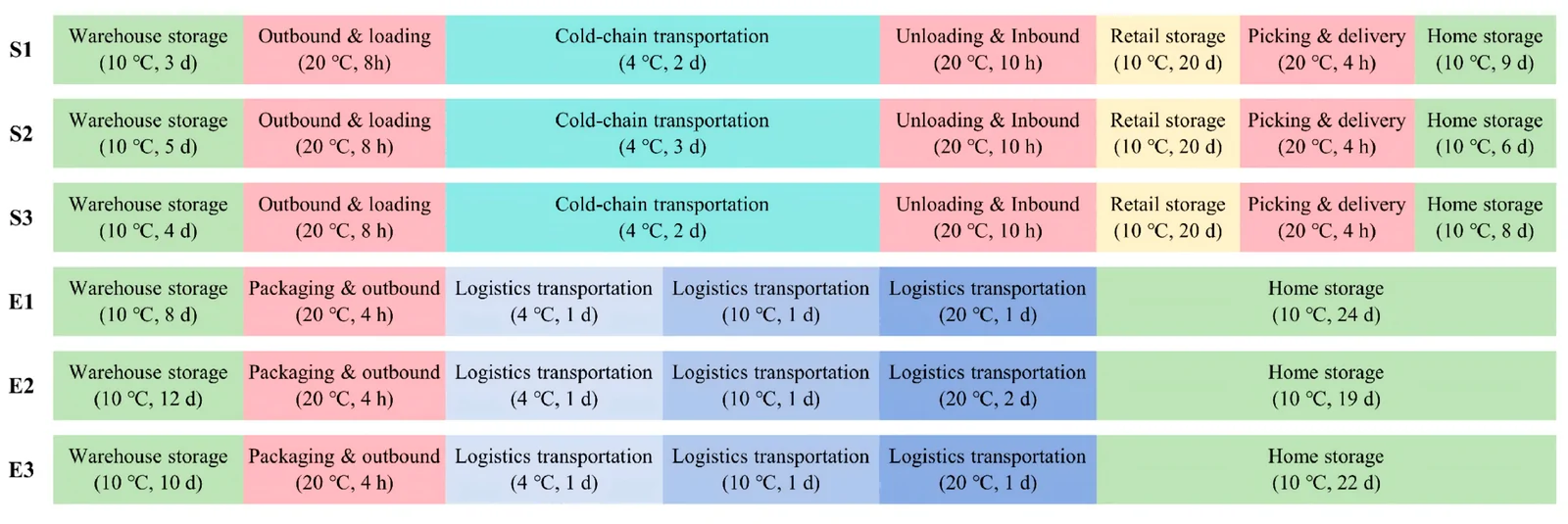
Temperature and time settings for different cold-chain transport modes of fermented soybean whey-based tofu.

Based on temperature monitoring of pre-packaged FSW-tofu during various distribution stages under different sales and transport modes (supermarket and e-commerce), the temperature variation range was determined to be 4–20 °C. In the supermarket mode, the temperature settings for each stage were as follows: warehouse storage at 10 °C, outbound and loading at 20 °C, cold-chain transportation at 4 °C, unloading and inbound at 20 °C, retail storage at 10 °C, picking and delivery at 20 °C, and home storage at 10 °C. In the e-commerce mode, the settings were as follows: warehouse storage at 10 °C, packaging and outbound at 20 °C, logistics transportation (4 °C on 1 d, 10 °C on 2 d, and 20 °C on 3–4 d), and household storage at 10 °C. Based on the temperature fluctuations in the different sales and cold-chain transport modes, six experimental groups were designed (S1, S2, S3 for supermarket mode; E1, E2, E3 for e-commerce mode), with storage temperature and time settings as shown in Figure 1. Samples from each group were randomly taken every 24 h of storage for the determination of TVC, PBC, textural properties, and WHC. Among them, groups S1, S2, E1, and E2 were used for the construction of the prediction model (training set, 4 out of 6 groups, approximately 67% of the total data), while groups S3 and E3 were used for model validation and real-time quality prediction algorithm testing (validation set, 2 out of 6 groups, approximately 33% of the total data).

### 2.5. Determination of TVC and PBC

A 5.0 g sample of FSW-tofu was aseptically weighed and homogenized with 45 mL saline (0.85%) for 5 min. All homogenates were serially diluted ten-fold with saline (0.85%). Then, 0.1 mL of the diluted sample was spread onto PCA and incubated at 37 °C for 48 h to determine the TVC [21].

Following the method of Na et al. [23], a 5.0 g FSW-tofu sample was mashed and placed into a conical flask containing 45 mL of sterile diluent and sterile glass beads. After homogenization and thorough shaking, ten-fold serial dilutions were prepared. An appropriate dilution (1 mL) was pipetted onto sterile Petri dishes, with two plates per dilution, and PCA was poured. After the agar solidified, the plates were inverted and incubated at 4 °C for 3 d to determine the PBC. Characteristic colonies from the plates were further verified by incubation at 37 °C; counts were considered valid only if no growth occurred at 37 °C.

### 2.6. Determination of Water-Holding Capacity

FSW-tofu samples were cut into 3 mm slices, and surface moisture was blotted with filter paper. Approximately 2.00 g of the slice (recorded as *m*_1_) was weighed and centrifuged at 15 °C and 6000 rpm for 10 min. After centrifugation, the sample mass (recorded as *m*_2_) was weighed [1]. WHC was calculated as the percentage of the sample mass after centrifugation relative to that before centrifugation, i.e., the ratio of *m*_2_ to *m*_1_. Each sample was measured in triplicate, and the average value was reported.

### 2.7. Determination of Textural Properties

Textural properties (hardness, springiness, and chewiness) of FSW-tofu were determined using a three-point sampling method with a texture analyzer. The measurement conditions were as follows: TPA (texture profile analysis) mode, a P35 cylindrical flat-bottom probe, preload stress of 0.05 N, compression and return speeds of 60 mm/min and 50 mm/min, respectively, and a 5 s interval between two compressions [6].

### 2.8. Statistical Analysis

All measurements were performed with three biological replicates (independent samples), and the data were expressed as mean ± standard deviation (SD). Given that each temperature trajectory was applied to a single group, the statistical inferences are primarily applicable to the specific temperature patterns tested. Data organization and preliminary processing were conducted using Origin software. For the mechanistic modeling, the Baranyi–Roberts primary model coupled with the Ratkowsky square-root secondary model was applied to describe non-isothermal microbial growth, while quality parameters (hardness, springiness, chewiness, and WHC) were first represented using parsimonious baseline response functions. Temperature-dependent and microbial-related deviations that were not explicitly represented by these baseline functions were subsequently captured by the machine learning residual-correction layer. Nonlinear regression was performed to estimate the kinetic parameters. The training dataset (groups S1, S2, E1, and E2) was used to calibrate the model, while the independent validation sets (groups S3 and E3) were strictly withheld for subsequent performance testing. It is important to emphasize that data splitting was performed at the trajectory/group level, not at the level of individual observations.

To improve prediction accuracy, a hybrid model was developed by incorporating random forest and gradient boosting tree algorithms for residual correction, with hyperparameters optimized via cross-validation on the training data. Model performance on the validation sets was evaluated using the coefficient of determination (R^2^), root mean square error (RMSE), mean absolute error (MAE), mean absolute percentage error (MAPE), and, for microbial counts, bias factor (B_f_) and accuracy factor (A_f_). All statistical analyses and model development were carried out using Python (version 3.9), with supplementary graphical work performed in Origin (version 9.1).

## 3. Results and Discussion

### 3.1. Effects of Different Logistics Modes on Microorganisms in FSW-Tofu

**Figure 2:**
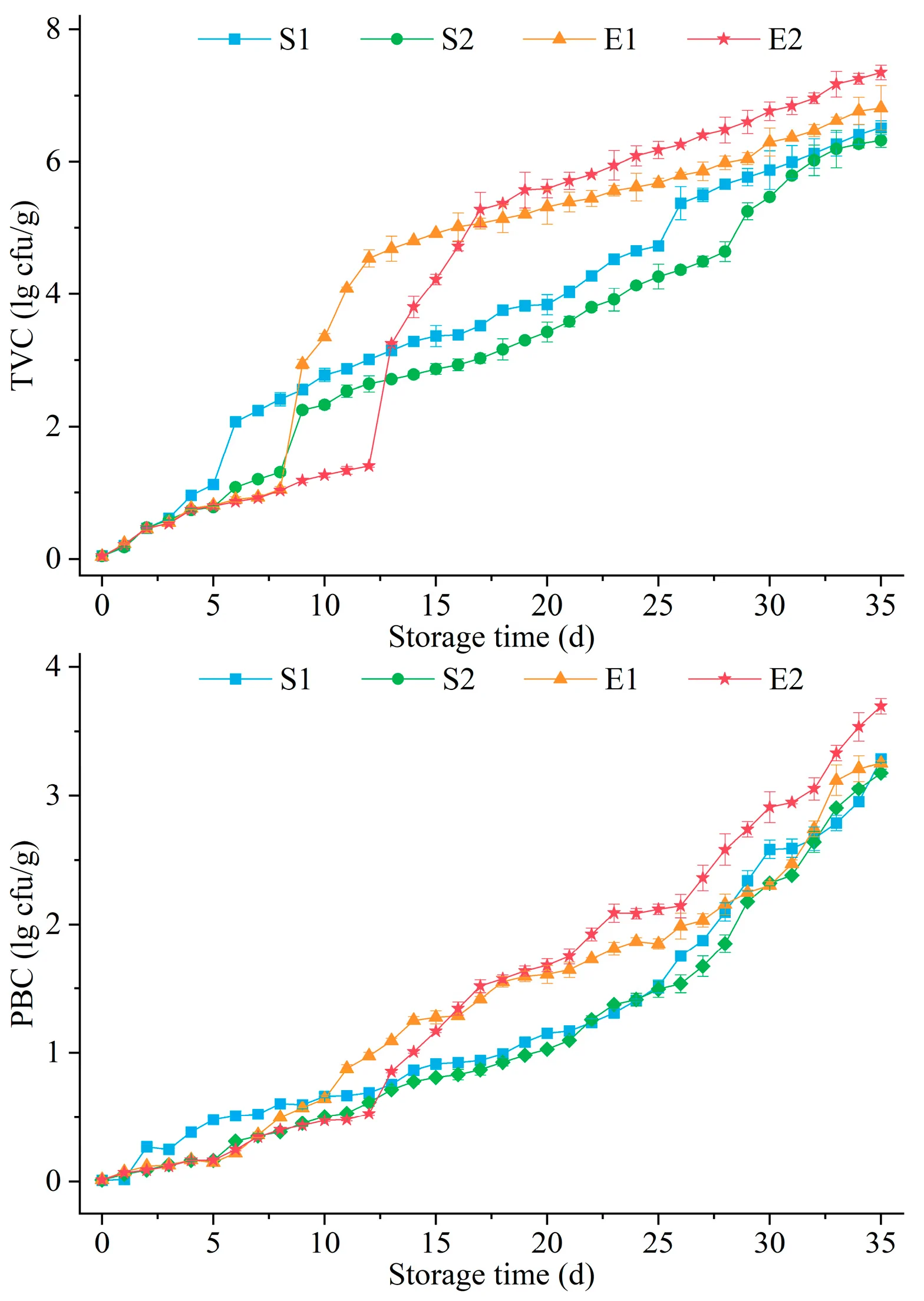
Changes in TVC and PBC in FSW-tofu under different logistics modes.

The changes in TVC and PBC of FSW-tofu under different cold-chain transport modes are presented in Figure 2. With increasing storage time, both TVC and PBC in the four experimental groups (S1, S2, E1, and E2) exhibited continuous upward trends, although the growth rates differed significantly among the logistics modes. After 35 d of storage, the TVC for groups S1, S2, E1, and E2 reached 6.51, 6.32, 6.82, and 7.35 lg CFU/g, respectively. Among these, group S2 showed the slowest TVC increase, whereas group E2 exhibited the most rapid growth, with counts significantly higher than those of the other three groups during the later storage period (after 25 d). In this study, the TVC threshold of 5 lg CFU/g was adopted as a TVC-based spoilage criterion, which specifies this value as the microbiological quality limit for commercial acceptability [6,21]. The four groups surpassed this threshold at approximately 24 d (S1), 27 d (S2), 20 d (E1), and 17 d (E2), respectively. The differences in TVC growth rates among the four groups were closely related to their respective cold-chain temperature fluctuation patterns. Group E2 experienced a high-temperature exposure (20 °C) on 4 d, and although the product was subsequently transferred to household storage at 10 °C, the earlier high temperature had already led to a substantial increase in the initial microbial load and shortened the lag phase of microbial growth. In contrast, group S2 experienced relatively small temperature fluctuations throughout the entire process (the temperature in each supermarket-mode stage was relatively stable), and the sustained low temperature effectively suppressed microbial proliferation. Kim et al. [24] reported that packaged tofu stored at 5, 13, and 23 °C showed faster increases in TVC at higher temperatures; the time from the end of shelf-life to reaching the initial spoilage level (10^6^ CFU/g) was 25 d at 5 °C, whereas it took only 1 d at 23 °C. This finding is consistent with the present observation that more severe temperature fluctuations led to faster microbial growth. Furthermore, Bang et al. [25] also demonstrated that during simulated last-mile delivery, the use of ice packs significantly mitigated temperature rise and microbial growth, thereby delaying the quality deterioration of tofu.

In terms of PBC changes, the four groups showed trends similar to those of TVC, albeit with relatively smaller overall increases. After 35 d of storage, PBC for S1, S2, E1, and E2 reached 3.29, 3.18, 3.26, and 3.70 lg CFU/g, respectively. Notably, during the later storage period (after 25 d), the PBC growth rate in group E2 accelerated markedly and became significantly higher than those of the other three groups. This phenomenon may be attributed to the fact that psychrophilic bacteria can maintain metabolic activity at low temperatures [26]; the larger microbial population resulting from prior high-temperature fluctuations, when subsequently transferred to low-temperature storage, allowed psychrophiles to gradually become the dominant flora. The PBC growth rate in group S1 during the mid-to-late storage period (15–35 d) was slightly higher than that in group S2, which may be related to the short-term temperature fluctuation (20 °C) experienced during the outbound and loading stage in group S1, indicating that even brief high-temperature exposure can exert cumulative effects on psychrophilic bacterial growth. While the present study did not perform species-level microbial identification, the differential growth patterns of TVC and PBC across temperature trajectories suggest that the dominant spoilage populations vary with temperature history, with psychrophilic bacteria becoming increasingly prevalent under prolonged low-temperature storage.

Soybean products are characterized by high water activity, low acidity, and high protein content, making them highly susceptible to rapid microbial growth and consequent spoilage [6,9]. Temperature fluctuations during cold-chain transportation of FSW-tofu accelerate microbial proliferation, and higher temperatures lead to faster microbial growth and more pronounced increases in TVC. Huang et al. [20] reported that FSW-tofu without added preservatives had a shelf-life of only 5 d at 4 °C. In the present study, after treatment with COS soaking solution and reverse-pressure sterilization, the initial TVC of the product was controlled at an extremely low level (<0.05 lg CFU/g), which significantly extended the microbial safety period, indicating that the pre-sterilization and bacteriostatic treatments were effective. Nevertheless, temperature fluctuations during cold-chain transport still caused notable microbial proliferation. Zeng et al. [21] also demonstrated that the addition of ε-polylysine could effectively delay the increase in TVC in pre-packaged FSW-tofu during refrigerated storage. In summary, the frequency and magnitude of temperature fluctuations during cold-chain transportation exerted a significant effect on microbial growth in FSW-tofu (*p* < 0.05); more severe fluctuations and longer exposure to elevated temperatures resulted in faster microbial proliferation and a shorter time for the product to reach the spoilage threshold. The product investigated in this study is a preserved FSW-tofu formulation containing 2 g/L COS and subjected to an 85 °C, 30 min thermal treatment; thus, the findings are specific to this product system and should not be directly extrapolated to untreated fresh FSW-tofu without further validation.

### 3.2. Effects of Different Logistics Modes on Textural Properties of FSW-Tofu

**Figure 3:**
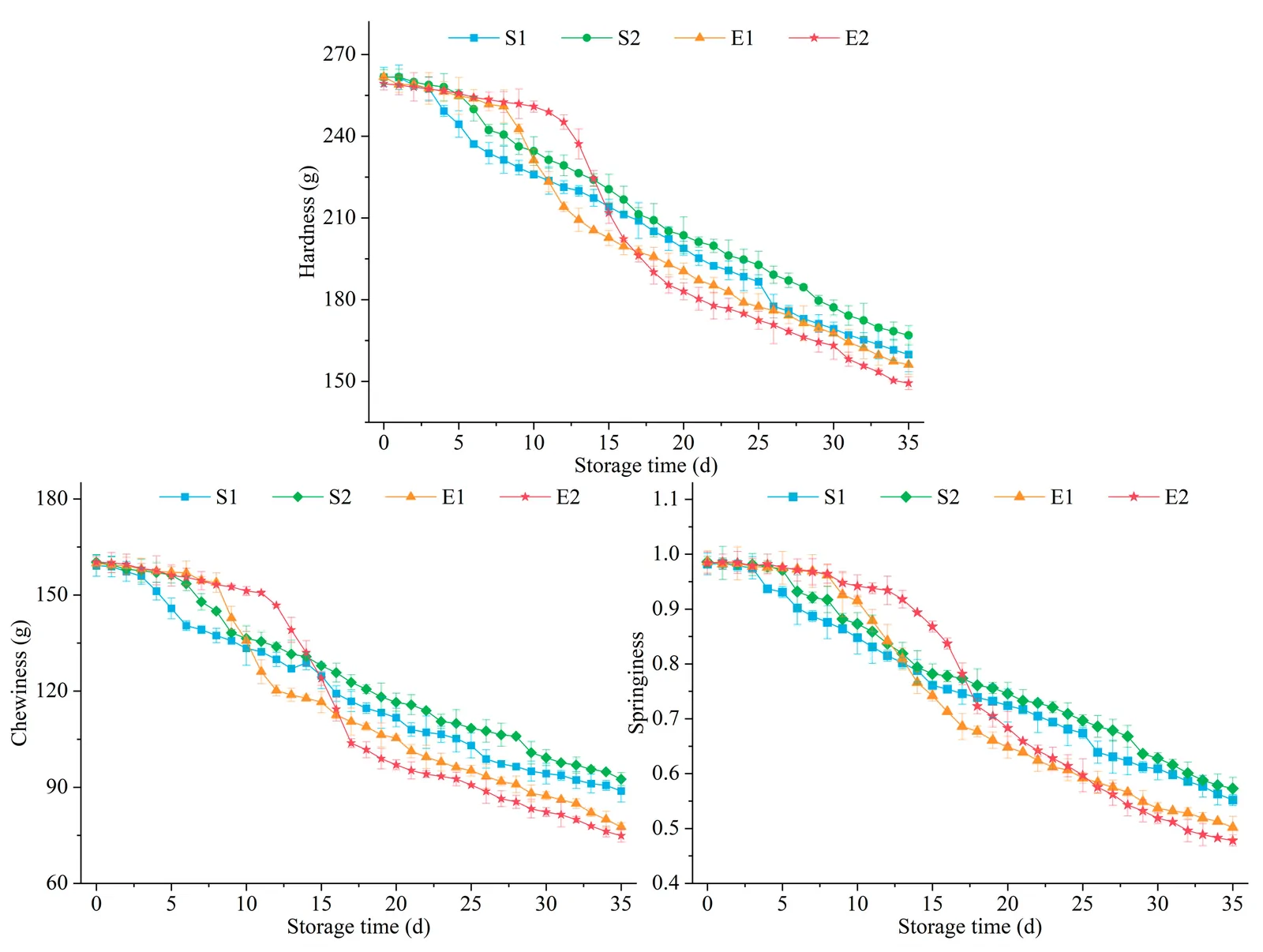
Changes in textural properties of FSW-tofu under different logistics modes.

The changes in textural properties (hardness, chewiness, and springiness) of FSW-tofu under different cold-chain transport modes are presented in Figure 3. With increasing storage time, the hardness, chewiness, and springiness of the four experimental groups all exhibited continuous declining trends, although the rates of decline differed significantly among the logistics modes. The initial hardness of groups S1, S2, E1, and E2 ranged from 259.26 to 261.77 g, and after 35 d of storage, the hardness decreased to 159.87 g, 166.89 g, 156.13 g, and 149.35 g, respectively, corresponding to reductions of 38.9%, 36.2%, 40.3%, and 42.4%. Among the four groups, group E2 showed the most pronounced decrease in hardness, particularly during the mid-to-late storage period (10–25 d), where its hardness values were significantly lower than those of the other three groups; group S2 exhibited the slowest decline and maintained relatively higher hardness throughout the entire storage period. Kim et al. [24] reported that the hardness of packaged tofu decreased sharply with increasing temperature and extended storage time, and the onset of visible decomposition coincided with the reduction in hardness. The decrease in tofu hardness is closely associated with the disruption of the protein gel network, because the gel structure of tofu is primarily maintained by soybean proteins through hydrophobic interactions, disulfide bonds, and hydrogen bonds, while the proteases produced by microbial metabolism degrade these proteins and destroy the gel network, leading to reduced hardness [5,27]. Group E2 experienced more severe temperature fluctuations during e-commerce logistics (from 4 °C to 10 °C to 20 °C), and the elevated temperatures accelerated microbial proliferation and protease secretion, thereby expediting the degradation of the protein gel network. In contrast, groups S1 and S2 under the supermarket mode experienced relatively constant temperatures (mainly in the range of 4–10 °C), which inhibited microbial activity and slowed protein degradation, thus resulting in better retention of hardness. In addition, protein oxidation is also an important factor contributing to the decline in tofu hardness. Recent studies have revealed the key role of protein oxidation in the deterioration of tofu gel during refrigerated storage, where it disrupts the gel network structure and causes textural quality degradation [28,29]. Research on seasoned dried tofu has also indicated that lipid-induced protein oxidation, which in turn alters the protein gel network structure, is a major mechanism underlying quality deterioration [9].

The changes in chewiness of FSW-tofu across all experimental groups followed a pattern similar to that of hardness. Group E2 exhibited the most substantial reduction, from an initial value of 160.27 g to 74.89 g, representing a decrease of 53.3%; group S2 showed the slowest decline, from 160.28 g to 92.54 g. Notably, the chewiness of groups E1 and E2 decreased at an accelerated rate during the mid-to-late storage period (after 15 d), which may be attributed to the enhanced microbial metabolic activity and accelerated protein degradation resulting from temperature fluctuations under the e-commerce mode. Huang et al. [20] found that the hardness and chewiness of FSW-tofu increased significantly during the first 3 d of refrigeration at 4 °C and then began to decrease. However, no such initial increase in hardness or chewiness was observed in the present study, which may be due to the reverse-pressure sterilization and COS soaking treatment applied in this experiment; the pre-sterilization effectively suppressed early microbial activity, causing the textural indicators to display a continuous decline from the outset of storage. Jiang et al. [30] also demonstrated that hardness and chewiness are key indicators affecting the quality changes in soybean products during storage.

The decrease in springiness of FSW-tofu followed the same trend as hardness and chewiness, with group E2 showing the most dramatic reduction and group S2 the most gradual decline. After 35 d of storage, the reductions reached 51.4% and 41.9%, respectively. The loss of springiness reflects the loss of elasticity in the tofu gel network, primarily caused by protein degradation, which reduces the cross-linking points within the gel network, thereby depriving the tofu of its original resilience and recoverability [9,31]. Compared with hardness, the decline in springiness was relatively more gradual, suggesting that the elastic structure of the tofu gel network is somewhat less sensitive to microbial degradation than the hardness indicator.

In summary, the textural properties of FSW-tofu exhibited a continuous deterioration trend during storage, and temperature fluctuation was the key factor accelerating textural degradation. This finding is consistent with the conclusion that temperature significantly affects hardness, adhesiveness, and chewiness in studies on *Premna microphylla* Turez jelly [32]. The deterioration of textural properties in FSW-tofu is primarily attributable to three causes: first, proteases produced by microbial metabolism directly degrade soybean proteins and destroy the gel network structure; second, free radicals generated from lipid oxidation induce protein oxidative denaturation, further exacerbating gel structure disruption; and third, increased moisture content and decreased protein content lead to reduced gel strength [33]. Therefore, maintaining temperature stability during cold-chain transport is critically important for preserving the textural quality of FSW-tofu.

### 3.3. Effects of Different Logistics Modes on Water-Holding Capacity of FSW-Tofu

**Figure 4:**
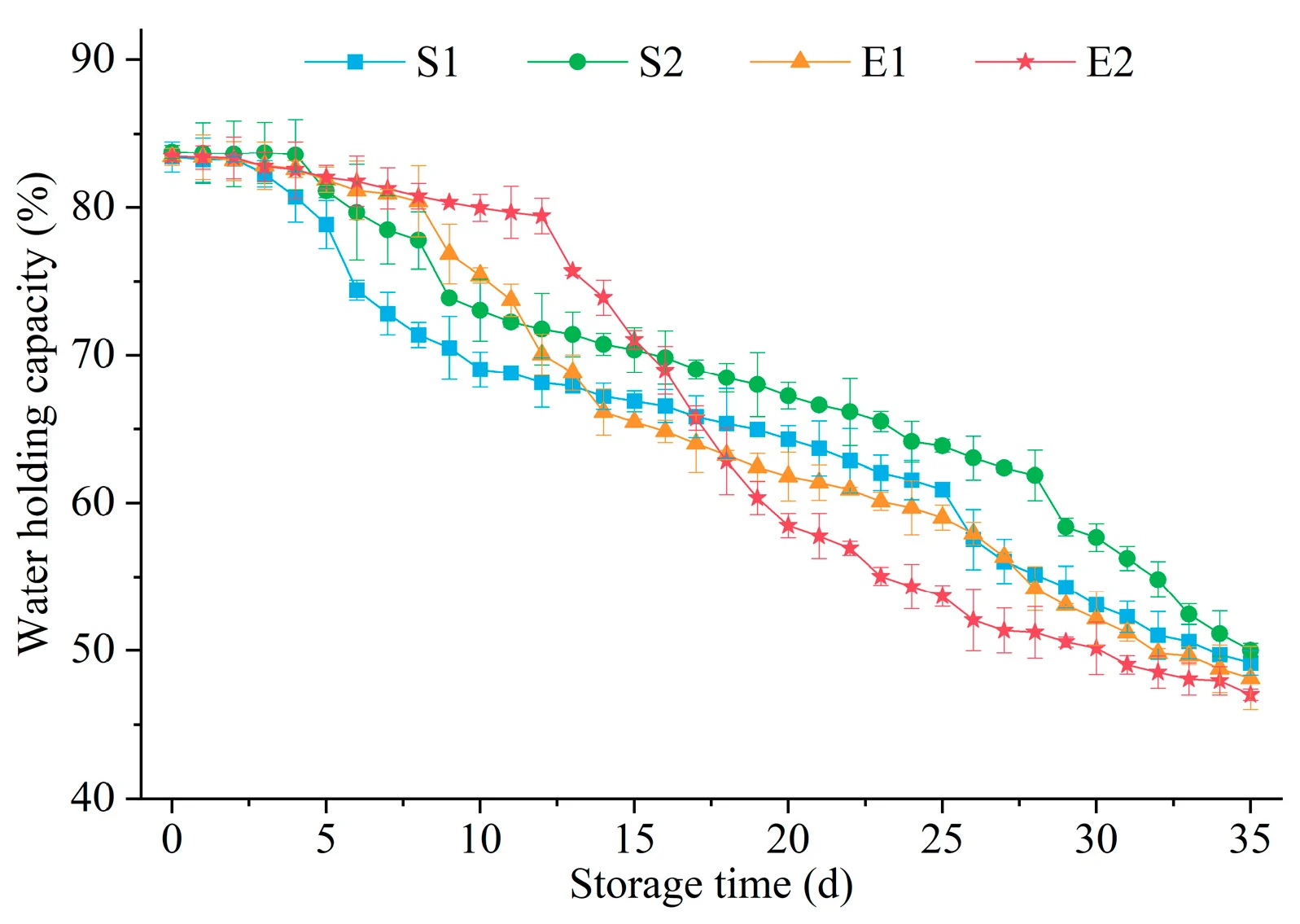
Changes in water-holding capacity of FSW-tofu under different logistics modes.

The changes in WHC of FSW-tofu under different cold-chain transport modes are presented in Figure 4. With increasing storage time, the WHC of all four experimental groups exhibited continuous declining trends, although the rates and patterns of decline differed significantly among the logistics modes. Among the four groups, group E2 showed the most pronounced decrease in WHC, with the decline rate accelerating notably during the later storage period (after 25 d), reaching 47.04% after 35 d of storage; group S2 exhibited the slowest decline and maintained relatively higher WHC throughout the entire storage period. This result indicates that the relatively stable low-temperature environment (4 to 10 °C) under the supermarket mode was beneficial for preserving the WHC of FSW-tofu, whereas the severe temperature fluctuations under the e-commerce mode (particularly the 20 °C high-temperature exposure) significantly accelerated the decline in WHC. Notably, group E1 showed a relatively slow decrease in WHC during the early-to-mid storage period (0–15 d), but the decline rate accelerated markedly during the later period (15–35 d), with the final WHC only slightly higher than that of group E2. This may be because group E1 experienced low-temperature environments of 4 °C and 10 °C during the initial 2 d of logistics transport, which inhibited microbial growth to some extent. However, the 20 °C high-temperature exposure on day 3 led to a substantial increase in the microbial population. Even after the product was subsequently transferred to household storage at 10 °C, the microorganisms maintained relatively high metabolic activity, continuously degrading proteins and disrupting the gel network structure. This phenomenon suggests that even a single episode of high-temperature exposure during cold-chain transport may exert long-term adverse effects on the WHC of FSW-tofu.

The WHC of tofu primarily depends on the physical entrapment of water by the protein gel network. When proteases produced by microbial metabolism degrade soybean proteins, the gel network structure is disrupted, and the water originally trapped within the network is gradually released, leading to a decline in WHC [34]. Studies have shown that during storage, soybean proteins undergo oxidative reactions, resulting in conformational changes and an overall decrease in gel strength and hardness [28]. In addition, free radicals generated from lipid oxidation can induce protein oxidative denaturation, alter protein molecular conformation, and further weaken the water-retaining capacity of the gel network [9,31]. Using low-field nuclear magnetic resonance, Zeng et al. [21] found that during refrigerated storage of FSW-tofu, immobilized water gradually transformed into free water, and the decline in WHC was closely related to the changes in water status. This finding is consistent with the observations in the present study, where the disruption of the protein gel network weakened the gel’s ability to retain water, resulting in a continuous decline in WHC. The decrease in WHC of FSW-tofu is the combined result of microbial protease degradation, protein oxidation, and water status transformation. Therefore, during cold-chain transportation and retail distribution, it is essential not only to control the storage temperature but also to pay close attention to the frequency and magnitude of temperature fluctuations in order to maximize the preservation of WHC and ensure product quality.

### 3.4. Construction of Microbial Growth Kinetic Models and Quality Response Models for FSW-Tofu

#### 3.4.1. Development and Evaluation of Microbial Growth Kinetic Models

To quantify the quality deterioration process of FSW-tofu under dynamic temperature conditions, the primary task was to accurately describe the growth behavior of the core variable, namely, microorganisms. In this study, the Baranyi–Roberts primary growth model was selected to describe the dynamic evolution of TVC and PBC. This model collectively characterizes the transition of microorganisms from the adaptation phase to the growth phase through the maximum specific growth rate (*μ_max_*), the maximum microbial count (*N_max_*), and the lag-adaptation state function (*Q*). However, during actual cold-chain transportation, the environmental temperature fluctuates dynamically over time, and *μ_max_* is not a constant. Therefore, the Ratkowsky power-law temperature response model was employed to describe the effect of temperature on *μ_max_*, and the real-time temperature was substituted into the Baranyi–Roberts model to achieve simulation of microbial growth under non-isothermal conditions. Furthermore, considering that the cold-chain temperature mainly fluctuates within the range of 4–20 °C, and to reduce the excessive amplification effect of short-term high-temperature exposure in the secondary model, the temperature response exponent was set to 1.5. The advantage of this combination lies in the fact that the Baranyi–Roberts model can effectively characterize the disturbance of temperature fluctuations on the microbial lag phase through the initial lag-state parameter (*h*_0_), while the Ratkowsky model provides a robust mathematical relationship between temperature and the maximum specific growth rate (*μ_max_*). The integration of these two models enables dynamic simulation of microbial growth under time-varying temperature conditions [35,36], with the specific calculations presented in Equations (1)–(4):

(1) $$\frac{\mathrm{dN}}{\mathrm{dt}}\;=\;\mu_{\max}(T)\;\times \;(1-\frac{N}{N_{\max}})\;\times \;\frac{Q}{1+Q}$$

(2) $$\frac{\mathrm{dQ}}{\mathrm{dt}}={\;\mu }_{\max}(T)\;\times \;Q\;\times \;(1-\frac{Q}{Q_{\max}})$$

(3) $$Q_{0}=\exp(-h_{0})$$

(4) $$\mu_{\max}(T)=b\;\times \;{\left(T-T_{\min}\right)}^{1.5}$$

In the above equations, *N* is the real-time microbial count; *N_max_* is the maximum microbial count; *N*_0_ is the initial microbial count; *t* is the storage time; *Q* is the lag-adaptation state function; *Q*_0_ is the initial lag-adaptation state; *Q_max_* is the saturation value of the lag state; *T* is the real-time–temperature; *μ_max_*(*T*) is the maximum specific growth rate at the real-time temperature; *h*_0_ is the initial lag-state parameter; *b* is the temperature response coefficient; *T_min_* is the minimum growth temperature of the microorganism; 1.5 is temperature response exponent.

Based on the measured data from the four training groups (S1, S2, E1, and E2), the key kinetic parameters of the models were first estimated by fitting (Table 1). The results showed that the growth model parameters for TVC and PBC (e.g., *N_max_*, *μ_max_*, *Q_max_*) all passed extremely significant tests (*p* < 0.001), and the confidence intervals of the parameters were relatively narrow, indicating good statistical stability and biological plausibility of the models. It is noteworthy that although the Ratkowsky model parameters *b_TVC* (0.0100) and *T_min__TVC* (0.4704) did not pass the significance tests (*p* = 0.092 and *p* = 0.426, respectively), this did not undermine the practical utility of the model. This is because these two parameters function as a synergistic pair in practical application; together, they define the temperature-response boundary for TVC growth, and their joint confidence interval effectively covers the temperature range encountered in actual cold-chain conditions. Compared with testing each parameter individually, the overall predictive performance of the model (as demonstrated in the validation results in Section 3.5) serves as a more critical indicator of its applicability. In addition, the *T_min_* for PBC was estimated at 5.66 °C, which is markedly higher than the 0.47 °C for TVC. This finding reveals the biological basis for the relatively high metabolic activity of psychrophilic bacteria at low temperatures and also explains why the proportion of PBC gradually increased during the later storage period (low-temperature stage) from a modeling perspective.

**Table 1:** Performance parameters of the prediction model on the training set.

Experimental Group	Indicators	B_f_	A_f_	R^2^	RMSE	MAPE
S1	TVC	1.0692	1.1695	0.9099	0.5594	19.1896
S1	PBC	1.1221	1.3009	0.9486	0.1989	44.0353
S1	WHC	1.0089	1.0213	0.9555	2.1084	2.1352
S1	Hardness	1.0042	1.0216	0.9670	5.6576	2.1492
S1	Chewiness	0.9832	1.0389	0.9438	5.2050	3.7737
S1	Springiness	0.9929	1.0306	0.9523	0.0288	3.0024
S2	TVC	1.2284	1.2303	0.8111	0.7956	27.2169
S2	PBC	1.2553	1.2616	0.9392	0.2210	33.5129
S2	WHC	0.9675	1.0381	0.9127	2.8459	3.6436
S2	Hardness	0.9753	1.0268	0.9510	6.6916	2.5895
S2	Chewiness	0.9476	1.0625	0.8734	7.6383	5.8205
S2	Springiness	0.9652	1.0425	0.9165	0.0376	4.0155
E1	TVC	0.9945	1.1907	0.9176	0.6381	21.7275
E1	PBC	1.0543	1.1473	0.9827	0.1231	20.3564
E1	WHC	1.0040	1.0168	0.9852	1.4167	1.6654
E1	Hardness	1.0114	1.0226	0.9716	5.9663	2.2613
E1	Chewiness	1.0071	1.0263	0.9775	4.1599	2.6271
E1	Springiness	1.0298	1.0401	0.9665	0.0321	4.0330
E2	TVC	1.1378	1.2354	0.9465	0.5985	29.3956
E2	PBC	1.0638	1.1834	0.9755	0.1746	24.1573
E2	WHC	1.0124	1.0429	0.9435	3.2523	4.2385
E2	Hardness	1.0074	1.0319	0.9624	7.8462	3.1538
E2	Chewiness	1.0088	1.0445	0.9673	5.7553	4.3864
E2	Springiness	1.0155	1.0499	0.9568	0.0396	4.9808

#### 3.4.2. Construction of Response Prediction Models for Quality Indicators

Microbial growth is not the endpoint of quality deterioration but rather the driving factor. To establish a quantitative relationship from the “cause” (microbial proliferation) to the “effect” (quality deterioration), response models for quality attributes were constructed based on experimental data. The core concept was to assume that the decline in quality parameters (hardness, springiness, chewiness, and WHC) is the cumulative result of microbial metabolic activity, as measured by total viable counts. Accordingly, response models for WHC (*W_t_*), hardness (*H_t_*), chewiness (*C_t_*), and springiness (*S_t_*) of FSW-tofu were established separately, with the specific calculations presented in Equations (5)–(8):

(5) $$W_{t}\;={\;W}_{\mathrm{eq}}+\;(W_{0}\;-\;W_{\mathrm{eq}})\;\times \;\exp(-k_{W}\sqrt{t}\;-\;k_{W2}t)$$

In the above equation, *W* is the WHC; *t* is the storage time; *W_eq_* is the equilibrium WHC; *W*_0_ is the initial WHC; *k_W_* is the primary WHC transfer coefficient; *k_W_*_2_ is the secondary WHC transfer coefficient.

(6) $$H_{t}=H_{0}\;\times \;\exp(-k_{H}{\;\times \;\log}_{10}(\frac{N_{t}}{N_{0}}))$$

In the above equation, *H* is the hardness; *t* is the storage time; *H*_0_ is the initial hardness; *k_H_* is the hardness decay coefficient; *N_t_* is the bacterial count at storage time *t*; *N*_0_ is the initial bacterial count.

(7) $$C_{t}=C_{0}-k_{C}{\;\times \;\log}_{10}(\frac{N_{t}}{N_{0}})-k_{\mathrm{CH}}\;\times \;(\frac{H_{0}-H_{t}}{H_{0}})$$

In the above equation, *C* is the chewiness; *t* is the storage time; *C*_0_ is the initial chewiness; *k_C_* is the bacterial influence coefficient; *N_t_* is the bacterial count at storage time *t*; *N*_0_ is the initial bacterial count; *k_CH_* is the hardness transfer coefficient; *H*_0_ is the initial hardness; *H_t_* is the hardness at storage time t.

(8) $$S_{t}=S_{0}-M_{N}(t)-M_{H}(t)-M_{W}(t)-M_{t}(t)-M_{T}(T)$$

In the above equation, *S* is the springiness; *t* is the storage time; *S*_0_ is the initial springiness; *M_N_(t)* is the microbial influence value at storage time *t*; *M_H_*(*t*) is the hardness influence value at storage time *t*; *M_W_*(*t*) is the water-holding capacity influence value at storage time *t*; *M_t_*(*t*) is the time influence value at storage time *t*; *M_T_*(*T*) is the temperature influence term at real-time temperature *T*.

The specific formulations of *M_N_*(*t*), *M_H_*(*t*), *M_W_*(*t*), *M_t_*(*t*), and *M_T_*(*T*) are given in Equations (9)–(13):

(9) $$M_{N}(t)\;=\frac{k_{S}}{1+\;\exp(-k_{S\_\mathrm{sig}}\;\times \;(\frac{N_{t}}{N_{0}}\;-\;3))}$$

In the above equation, *N_t_* is the bacterial count at storage time *t*; *N*_0_ is the initial bacterial count; *k_S_* is the influence coefficient of bacterial count on springiness; *k_S_sig_* is the shape parameter of the sigmoid function.

(10) $$M_{H}(t)=k_{SH}\;\times \;(\frac{H_{0}-H_{t}}{H_{0}})\;\times \;[1+0.5\;\times \;(\frac{H_{0}-H_{t}}{H_{0}})]$$

In the above equation, *H_t_* is the hardness at storage time t; *H*_0_ is the initial hardness; *k_SH_* is the transfer coefficient between hardness and springiness.

(11) $$M_{W}(t)=k_{SW}\;\times \;(\frac{W_{0}-W_{t}}{W_{0}})\;\times \;(1+0.3\;\times \sqrt{\frac{W_{0}-W_{t}}{W_{0}}})$$

In the above equation, *W_t_* is the WHC at storage time *t*; *W*_0_ is the initial WHC; *k_SW_* is the transfer coefficient between WHC and springiness.

(12) $$M_{t}(t)=k_{S\text{\_}\mathrm{time}}\;\times \;\ln(1+\frac{t}{12})$$

In the above equation, *k_S_time_* is the influence coefficient of time on springiness; *t* is the storage time.

(13) $$M_{T}(T)=k_{S\text{\_}\mathrm{temp}}\;\times \;[\exp(0.1\;\times \;(T-4))-1]$$

In the above equation, *k_S_temp_* is the influence coefficient of temperature on springiness; *T* is the real-time temperature.

It should be noted that the WHC response model was formulated exclusively as a function of storage time, in contrast to the other quality response models that incorporate microbial or textural variables. This decision was based on two considerations. First, WHC reflects the progressive physical disruption of the protein gel network caused by the cumulative action of extracellular proteases over time, which, unlike microbial count itself, does not follow an exponential growth pattern but rather a gradual, time-dependent deterioration trajectory [34,36]. Second, preliminary analysis revealed that microbial count alone was a poor predictor of WHC decline (R^2^ = 0.41), whereas time-based fitting provided excellent performance (R^2^ = 0.94). This is biologically plausible because the gel network degradation that governs WHC is a cumulative process driven by the total protease activity accumulated over the entire storage period, which correlates more strongly with time than with instantaneous microbial counts. Temperature effects on WHC are indirectly embedded in the model through the experimental grouping design, as the rate parameters *k_W_* and *k_W_*_2_ were separately estimated for each temperature trajectory in the training dataset, capturing the differential deterioration rates caused by distinct temperature histories. Thus, while the WHC model is structurally simpler than the other quality response models, it remains consistent with the underlying physical mechanism and provides accurate predictions for the temperature ranges tested.

The model construction results indicated that each quality response model exhibited good fitting ability to the training data (Table 2). Taking the hardness response model as an example, its decay coefficient *k_H_* was 0.1967, implying that for each logarithmic increase in TVC, hardness decreased by approximately 19.7%, which is highly consistent with the declining trend of hardness shown in Figure 3. The chewiness response model further revealed its multi-factor-driven deterioration characteristics: it was affected not only directly by TVC (*k_C_* = 9.77) but also indirectly through the decline in hardness (*k_CH_* ≈ 1.0), indicating that the disruption of the gel network structure was the primary cause of chewiness loss. The springiness response model, on the other hand, constructed a more comprehensive framework that integrated multiple influences, including microbial counts, hardness, WHC, storage time, and temperature. Although the significance levels of the two parameters *k_S_temp_* and *k_S_sig_* in this model (*p* = 0.159 and *p* = 0.104) did not reach the conventional statistical criteria, their inclusion as components of the model system helped to improve the overall robustness of the predictions. Overall, the quality response models successfully converted the “invisible” microbial growth into “visible” changes in texture and WHC, thereby providing a quantitative tool for model-based quality monitoring in subsequent applications.

**Table 2:** Kinetic parameters of the microbial growth kinetic models and quality response models.

Models	Parameters	Fitted Values	*t*	Standard Error	95% Confidence Interval	*p* Values	MSE
Baranyi–Roberts kinetic models	*N_max__TVC*	7.9130	26.8575	0.2946	[7.32941, 8.4965]	<0.001	0.5178
Baranyi–Roberts kinetic models	*μ_max__TVC*	0.0600	5.0914	0.0118	[0.03666, 0.083344]	<0.001	
Baranyi–Roberts kinetic models	*Q_max__TVC*	0.8180	6.9410	0.1179	[0.584583, 1.05142]	<0.001	
Ratkowsky kinetic models	*b_TVC*	0.0100	1.6982	0.0059	[−0.001664, 0.021678]	0.092	
Ratkowsky kinetic models	*T_min__TVC*	0.4704	0.7983	0.5893	[−0.696667, 1.63752]	0.426	
Baranyi–Roberts kinetic models	*h_0__TVC*	2.4274	4.1194	0.5893	[1.26029, 3.59448]	<0.001	
Baranyi–Roberts kinetic models	*N_max__PBC*	4.1824	14.1956	0.2946	[3.59887, 4.76597]	<0.001	0.0440
Baranyi–Roberts kinetic models	*μ_max__PBC*	0.0500	4.2425	0.0118	[0.026657, 0.073341]	<0.001	
Baranyi–Roberts kinetic models	*Q_max__PBC*	1.4714	12.4851	0.1179	[1.23796, 1.7048]	<0.001	
Ratkowsky kinetic models	*b_PBC*	0.0164	2.7901	0.0059	[0.00477, 0.028112]	0.006	
Ratkowsky kinetic models	*T_min__PBC*	5.6628	9.6102	0.5893	[4.49575, 6.82994]	<0.001	
Baranyi–Roberts kinetic models	*h*_0_ *_PBC*	2.4806	4.2097	0.5893	[1.31349, 3.64768]	<0.001	
WHC response prediction model	*W*_0_	79.9317	67.8243	1.1785	[77.5975, 82.2659]	<0.001	7.4802
WHC response prediction model	*W_eq_*	55.0356	46.6993	1.1785	[52.7014, 57.3698]	<0.001	
WHC response prediction model	*k_W_*	0.0166	14.0752	0.0012	[0.014254, 0.018922]	<0.001	
WHC response prediction model	*k_W_*_2_	0.0026	8.9457	0.0003	[0.002052, 0.003219]	<0.001	
Hardness response prediction model	*H*_0_	269.2873	22.8498	11.7851	[245.945, 292.629]	<0.001	52.4379
Hardness response prediction model	*k_H_*	0.1967	33.3884	0.0059	[0.185072, 0.208414]	<0.001	
Chewiness response prediction model	*C*_0_	132.9134	15.0374	8.8388	[115.407, 150.42]	<0.001	41.9049
Chewiness response prediction mode	*k_C_*	9.7679	33.1533	0.2946	[9.18434, 10.3514]	<0.001	
Chewiness response prediction mode	*k_CH_*	0.9770	33.1594	0.0295	[0.918613, 1.03532]	<0.001	
Springiness response prediction mode	*S*_0_	0.9443	16.0249	0.0589	[0.827565, 1.06098]	<0.001	0.0010
Springiness response prediction mode	*k_S_*	0.0324	5.5024	0.0059	[0.020753, 0.044094]	<0.001	
Springiness response prediction mode	*k_SH_*	0.0677	5.7445	0.0118	[0.044358, 0.091042]	<0.001	
Springiness response prediction mode	*k_SW_*	0.0779	26.4445	0.0029	[0.072077, 0.083748]	<0.001	
Springiness response prediction mode	*k_S_time_*	0.0392	33.2809	0.0012	[0.036888, 0.041556]	<0.001	
Springiness response prediction mode	*k_S_temp_*	0.0008	1.4189	0.0006	[−0.000331, 0.002003]	0.159	

### 3.5. Establishment and Validation of Dynamic Prediction Models for FSW-Tofu Quality

#### 3.5.1. Construction of the Hybrid Mechanistic-Machine Learning Model

Purely mechanism-based models often suffer from insufficient prediction accuracy when confronted with complex, non-linear actual cold-chain fluctuations [18,37]. To address this issue, a novel hybrid modeling strategy was introduced in this study, incorporating a machine learning residual-correction mechanism following the principle of “mechanistic model first for preliminary prediction, then machine learning for bias correction”. Specifically, the mechanistic models (microbial kinetics and quality responses) established in Section 3.4 were first used to generate preliminary predictions for the quality indicators, providing baseline values. Subsequently, a composite machine learning model, with random forest and gradient boosting trees as the core algorithms, was constructed. Its input feature matrix (X) included not only basic variables such as time and temperature, but also mechanistic model predictions, temperature change rates, and interaction terms among the various quality indicators. The task of this machine learning model was to learn the residuals (i.e., observed value minus mechanistic prediction) between the mechanistic predictions and the measured values, and finally to output the corrected final predictions (Equation (14)). This design preserves the biological interpretability of the mechanistic models while leveraging the powerful non-linear fitting capability of machine learning to effectively capture the complex fluctuation effects that mechanistic models fail to describe.

(14) $${\hat{y}}_{j}=f_{\mathrm{kin},j}(t,T)+g_{j}(X)$$

In the above equation, *ŷ_j_* is the final predicted value for the *j*-th quality indicator; *f_kin,j_*(*t,T*) is the kinetic or quality response model prediction for the *j*-th indicator at storage time t and real-time temperature *T*; *g_j_*(*X*) is the machine learning residual-correction function for the *j*-th indicator; *X* is the feature matrix composed of time, temperature, mechanistic predictions, interaction terms, and rates of change.

#### 3.5.2. Model Prediction Performance Evaluation and Validation

The prediction performance of the models was evaluated using MSE, RMSE, MAPE, B_f_, A_f_, and R^2^. The specific calculations are presented in Equations (15)–(20).

(15) $$\mathrm{MSE}=\frac{1}{n}\Sigma_{i=1}^{n}{\left(y_{i}-{\hat{y}}_{i}\right)}^{2}$$

(16) $$\mathrm{RMSE}=\sqrt{\frac{1}{n}\Sigma_{i=1}^{n}{\left(y_{i}-{\hat{y}}_{i}\right)}^{2}}$$

(17) $$\mathrm{MAPE}=\frac{100}{n}\Sigma_{i=1}^{n}|\frac{y_{i}-{\hat{y}}_{i}}{y_{i}}|\%$$

(18) $$B_{f}=10^{\frac{1}{n}\Sigma_{i=1}^{n}\log_{10}(\frac{{\hat{y}}_{i}}{y_{i}})}$$

(19) $$A_{f}=10^{\frac{1}{n}\Sigma_{i=1}^{n}|\log_{10}(\frac{{\hat{y}}_{i}}{y_{i}})|}$$

(20) $$R^{2}\;=1-\frac{\Sigma_{i=1}^{n}{\left(y_{i}-{\hat{y}}_{i}\right)}^{2}}{\Sigma_{i=1}^{n}{(y_{i}-\overset{-}{y})}^{2}}$$

In the above equations, *n* is the number of samples; *i* is the sample index; *y_i_* is the *i*-th actual observed value; *ŷ_i_* is the *i*-th predicted value.

**Figure 5:**
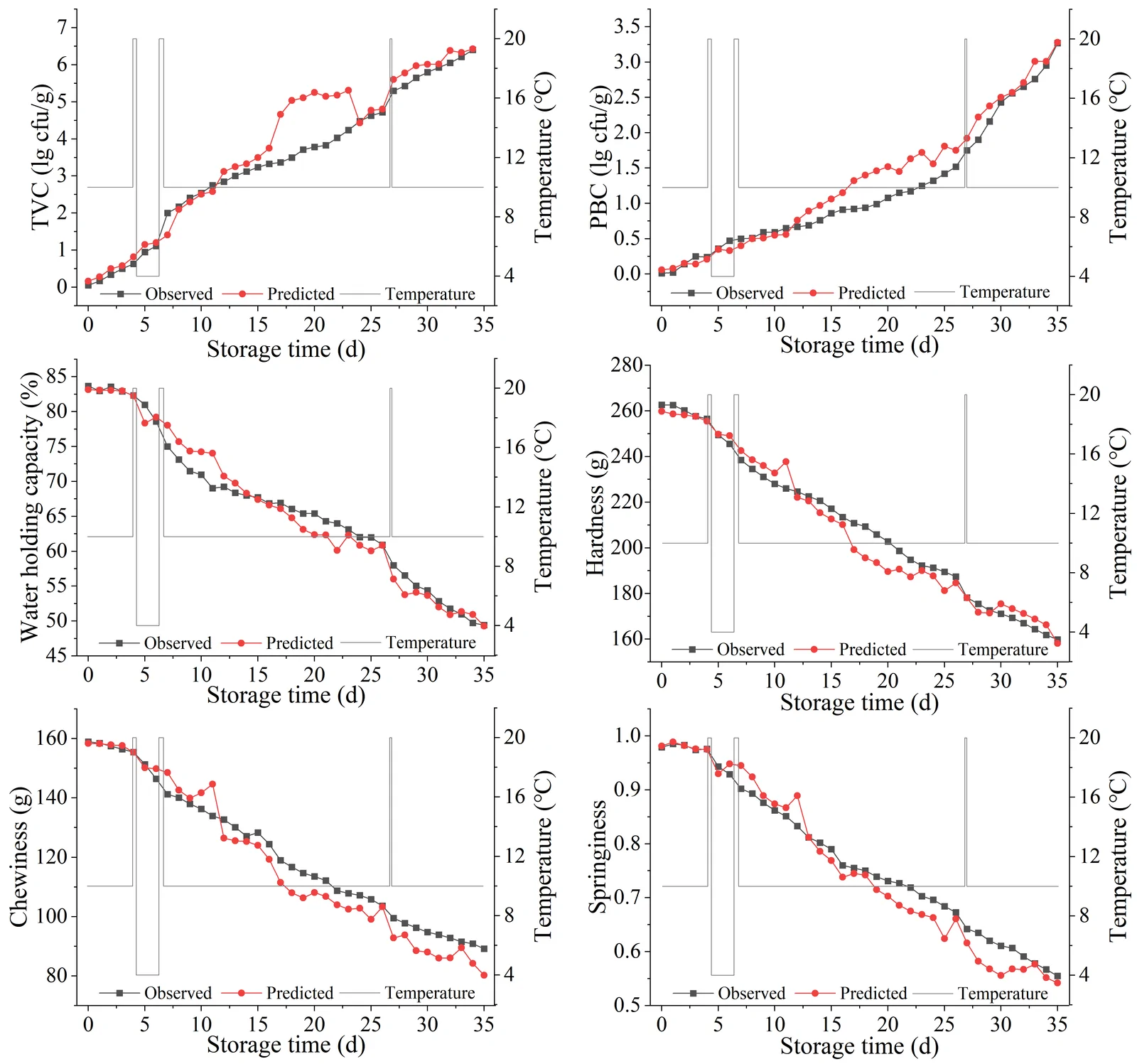
Fitting results of measured and predicted values for microbial and quality indicators of FSW-tofu in validation set S3.

**Figure 6:**
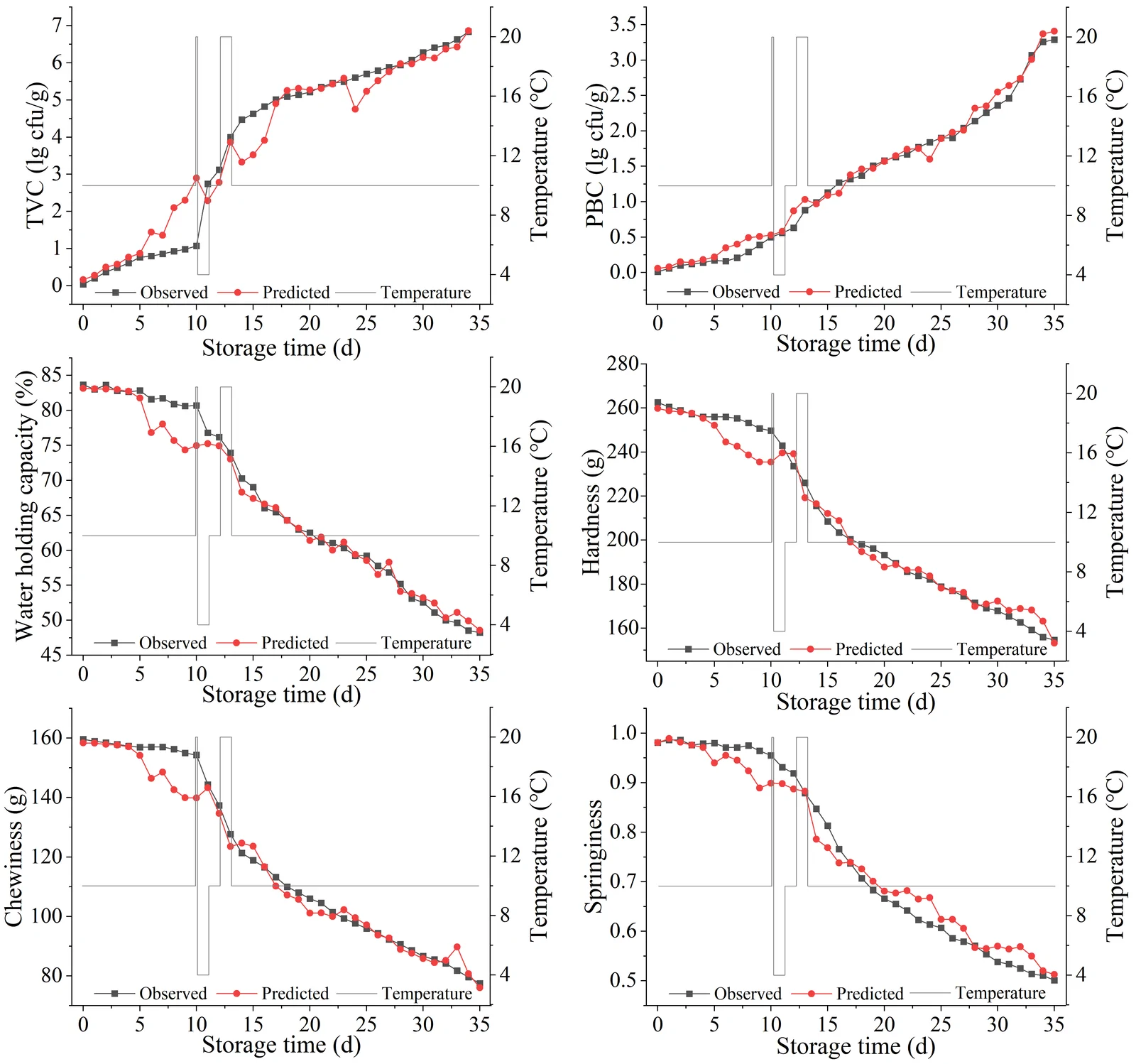
Fitting results of measured and predicted values for microbial and quality indicators of FSW‑tofu in validation set E3.

To comprehensively validate the generalization ability of the constructed hybrid model (mechanistic and machine learning), it was applied to the completely independent validation datasets S3 and E3, which had not been used in model training. As shown in Table 3 and Figure 5 and Figure 6, the model accurately captured the overall trends of TVC and PBC changes. In groups S3 and E3, the R^2^ values for TVC predictions were 0.8924 and 0.9395, respectively, while those for PBC were 0.9236 and 0.9863, indicating that the model could explain the vast majority of the data variability. The relatively higher MAPE values observed during early storage (when microbial concentrations were low) reflect the known sensitivity of MAPE to small denominators rather than systematic prediction errors; this is a well-recognized limitation in predictive microbiology and does not compromise the overall model validity. More importantly, the RMSE values for TVC (0.5594–0.7956 lg CFU/g) and PBC (0.1231–0.2210 lg CFU/g), together with B_f_ values close to one (0.9476–1.2553) and A_f_ values within acceptable ranges (1.0389–1.3009), collectively demonstrate satisfactory predictive accuracy.

For the quality indicators, the model performed exceptionally well in predicting hardness, springiness, chewiness, and WHC. In both validation sets, the R^2^ values for all quality indicators exceeded 0.93, and RMSE values remained at relatively low levels. For instance, the MAPE for hardness and springiness ranged between 2.0% and 3.7%, which was much lower than the error level for the microbial indicators. This demonstrates that the quality response models, driven by microbial predictions, provide a robust and accurate transmission of predictions.

Furthermore, when comparing the performance metrics of the training set (Table 1) and the validation set (Table 3), no significant decline was observed in R^2^, RMSE, B_f_, or A_f_ for any of the indicators. This indicates that the model did not suffer from overfitting and successfully learned the universal patterns of quality deterioration induced by temperature fluctuations, showing good adaptability to different cold-chain transport modes.

**Table 3:** Performance parameters of the prediction model on the validation set.

Experimental Group	Indicators	B_f_	A_f_	R^2^	RMSE	MAPE
S3	TVC	1.1546	1.1880	0.8924	0.6209	22.1776
S3	PBC	1.1868	1.3102	0.9236	0.2403	44.1510
S3	WHC	0.9955	1.0223	0.9648	1.8973	2.2011
S3	Hardness	0.9914	1.0240	0.9636	6.0264	2.3455
S3	Chewiness	0.9695	1.0448	0.9379	5.5451	4.2784
S3	Springiness	0.9789	1.0342	0.9513	0.0294	3.2904
E3	TVC	1.1414	1.2466	0.9395	0.5801	32.1348
E3	PBC	1.1737	1.2008	0.9863	0.1138	27.6173
E3	WHC	0.9905	1.0209	0.9691	2.1410	2.0364
E3	Hardness	0.9956	1.0213	0.9722	6.1857	2.0954
E3	Chewiness	0.9861	1.0285	0.9665	5.3235	2.7689
E3	Springiness	1.0069	1.0375	0.9676	0.0326	3.7017

### 3.6. Construction of Rapid Texture-Based Assessment Indicators Anchored to TVC-Based Spoilage Thresholds

The preceding models have accurately described the complete chain of how “temperature fluctuations induce microbial growth, which in turn leads to quality deterioration”. However, in practical industrial applications, the timeliness and complexity of microbial testing remain major bottlenecks. Therefore, this section aims to extract simplified indicators from the established models that can be used for rapid on-site assessment. In this study, a TVC of 5 lg CFU/g was established as the TVC-based spoilage threshold for FSW-tofu transitioning from “acceptable” to “unacceptable” [6,21], a threshold that carries important quality and commercial significance. During model validation, we extracted the corresponding measured quality data at the time when TVC first reached or exceeded this threshold in validation groups S3 and E3 (Table 4).

Analysis of the data in Table 4 reveals a seemingly counter-intuitive phenomenon: the textural properties, including hardness, springiness, and chewiness of FSW-tofu in group E (e-commerce mode) were significantly superior to those in group S (supermarket mode) under the condition of an identical TVC threshold (approximately 5.0–5.3 lg CFU/g). For example, at a TVC of 5.01 lg CFU/g in group E3, the springiness was 0.737, whereas at a TVC of 5.30 in group S3, the springiness was only 0.642. This does not imply that the e-commerce mode better preserves quality; rather, it reveals a key mechanism: under rapid deterioration modes, microbial counts and textural changes are not fully synchronized. Samples in group E experienced severe temperature fluctuations, allowing microorganisms to grow rapidly and cross the threshold within a relatively short period (16–17 d), during which degradation of the protein gel network was still in its early stages and texture remained relatively intact. In contrast, samples in group S were subjected to sustained mild low-temperature stress, providing microorganisms with a longer period (27 d) to secrete proteases and conduct a more thorough “chronic” hydrolysis of the protein network, resulting in more severe textural damage by the time equivalent bacterial counts were reached.

This finding carries important practical implications. It demonstrates that a single texture value cannot serve as a universally applicable “rigid” indicator of quality change. This phenomenon also indirectly suggests that the composition of the spoilage microbiota, rather than total count alone, may influence the extent of textural degradation at equivalent TVC levels, a hypothesis that will be addressed in our ongoing studies. However, through the predictive models established in this study, it is possible to dynamically calculate the theoretical texture values corresponding to the time when TVC reaches 5 lg CFU/g, based on the specific time–temperature history of a given batch, thereby providing a “dynamic” rapid assessment reference for that batch. For instance, for products that have experienced a mild cold-chain pattern similar to group S, when springiness declines to approximately 0.64, caution should be exercised as the TVC-based spoilage threshold may have been reached; whereas for products that have experienced greater fluctuations similar to group E, the threshold is reached when springiness is still at approximately 0.73.

Therefore, the “rapid assessment indicator” constructed in this study is not a set of fixed numerical values, but rather a dynamic determination method coupled with the predictive model. Quality control personnel in enterprises can input real-time monitored texture data into the trained hybrid model to back-calculate the estimated current TVC or to predict the remaining time before the microbial threshold is reached at the given texture level. This approach is more scientific and reliable than simply relying on empirical critical texture values and provides an efficient decision-support tool for quality monitoring in cold-chain logistics. Future work will further strengthen the present findings by incorporating species-level microbial identification to elucidate the composition of spoilage microbiota under different temperature trajectories, as well as metabolite profiling to directly characterize the biochemical pathways of quality deterioration. Additional temperature trajectory replications and production batches would also enhance the statistical robustness and generalizability of the model.

## 4. Conclusions

This study addressed the non-isothermal quality deterioration problem of pre-packaged FSW-tofu (containing 2 g/L COS, sterilized at 85 °C, 0.1 MPa, 30 min) during cold-chain transportation. The findings and the predictive model developed herein are specifically applicable to this preserved product formulation and processing conditions; extrapolation to untreated fresh FSW-tofu or products with different preservation treatments requires further validation. By simulating two typical logistics modes (supermarket and e-commerce), the differential driving effects of temperature fluctuation frequency and amplitude on microbial proliferation and textural degradation were systematically revealed. Through the integration of the Baranyi–Roberts–Ratkowsky non-isothermal kinetic model with a machine learning residual-correction algorithm, a hybrid dynamic prediction model covering TVC, PBC, hardness, springiness, chewiness, and WHC was successfully constructed. Validation results demonstrated that the model exhibited good generalization ability on independent datasets (R^2^ > 0.9). On this basis, using TVC = 5 lg CFU/g as the TVC-based spoilage threshold, a dynamic texture-based rapid assessment method specific to given temperature histories was further proposed, effectively overcoming the limitations of conventional fixed thresholds in practical applications. This study provides a scientifically sound and practically applicable new tool for cold-chain quality monitoring and rapid freshness assessment of FSW-tofu and similar fresh soybean products. Moreover, the results highlight that temperature stability is critical for preserving quality, and the established hybrid model can serve as a decision-support tool for enterprises to predict remaining shelf-life based on real-time temperature monitoring data.

## Figures and Tables

**Table 4:** Quality parameters of FSW-tofu at the TVC spoilage threshold in each experimental group.

Experimental Group	Storage Time (d)	TVC (lg CFU/g)	PBC (lg CFU/g)	Springiness	Chewiness (g)	Hardness (g)	WHC (%)
S1	26	5.37	1.75	0.639	98.82	177.56	57.54
S2	29	5.25	2.18	0.636	100.81	179.65	58.38
S3	27	5.30	1.75	0.642	99.47	178.16	57.97
E1	16	5.02	1.29	0.713	112.50	199.55	64.84
E2	17	5.28	1.52	0.782	103.76	196.14	65.74
E3	17	5.01	1.32	0.737	113.22	200.39	65.49

## Data Availability

The original contributions presented in this study are included in the article. Further inquiries can be directed to the corresponding author.
